# Developing Academic Advisors and Competence Committees members: A community approach to developing CBME faculty leaders

**DOI:** 10.36834/cmej.68181

**Published:** 2020-03-16

**Authors:** Eleftherios Soleas, Damon Dagnone, Denise Stockley, Kendall Garton, Richard van Wylick

**Affiliations:** 1 Queens University, Ontario, Canada

## Abstract

**Introduction:**

Implementing competency-based medical education (CBME) at the institutional level poses many challenges including having to rapidly enable faculty to be facilitators and champions of a new curriculum which utilizes feedback, coaching, and models of programmatic assessment. This study presents the necessary competencies required for Academic Advisors (AA) and Competence Committee (CC) members, as identified in the literature and as perceived by faculty members at Queen’s University.

**Methods:**

This study integrated a review of available literature (n=26) yielding competencies that were reviewed by the authors followed by an external review consisting of CBME experts (n=5). These approved competencies were used in a cross-sectional community consultation survey distributed one year before (n=83) and one year after transitioning to CBME (n=144).

**Findings:**

Our newly identified competencies are a useful template for other institutions. Academic Advisor competencies focused on mentoring and coaching, whereas Competence Committee member’s competencies focused on integrating assessments and institutional policies. Competency discrepancies between stakeholder groups existing before the transition had disappeared in the post-implementation sample.

**Conclusions:**

We found value in taking an active community-based approach to developing and validating faculty leader competencies sooner rather than later when transitioning to CBME. The evolution of Competence Committees members and Academic Advisors requires the investment of specialized professional development and the sustained engagement of a collaborative community with shared concerns.

## Abstract (fr)

**Contexte:**

La mise en œuvre d’une formation médicale fondée sur les compétences (FMFC) au niveau institutionnel pose de nombreux défis, y compris de devoir permettre au corps professoral de devenir rapidement des facilitateurs et des champions d’un nouveau cursus qui fait appel à la rétroaction, à l’accompagnement et à l’évaluation programmatique. Cette étude présente les compétences nécessaires requises pour les conseillers pédagogiques(CP) et les membres des comités des compétences (CC), tel qu’identifié dans la littérature et comme perçues par le corps professoral à l’Université Queen.

**Méthodes:**

Cette étude a intégré une recension des écrits disponibles (n = 26) identifiantdes compétences, qui ont été évaluéespar les auteurs, suivie d’une évaluation externe composée d’experts de la FMFC (n = 5). Ces compétences approuvées ont été utilisées dans une consultation communautaire transversale distribuée une année avant(n = 83) et une année après la transition vers la FMFC (n = 144).

**Résultats:**

Nos compétences nouvellement déterminées représentent un modèle utile pour d’autres institutions. Les compétences d’un conseiller pédagogiquesont axées sur le mentorat et l’accompagnement, alors que les compétences des membres des comités des compétences sont axées sur l’intégration des évaluations et des politiques institutionnelles. Les divergences dans les compétences entre les parties prenantes existants avant la transition avaient disparu dans l’échantillon qui a suivi la mise en œuvre.

**Conclusions:**

Nous avons jugé utile d’adopter une approche active fondée sur lacommunauté pour élaborer et valider les compétences du corps professoral en position de leadership plus tôt que tard dans la transition vers la FMFC. L’évolution des membres des comités de compétences et des conseillers pédagogiquesnécessite un investissement dans un développement professoral spécialisé et un engagement soutenu d’une communauté collaborative qui présente des préoccupations communes.

## Introduction

Implementing competency-based medical education (CBME) at the institutional level poses many challenges.^1^^–^^3^ One of these is having to rapidly enable faculty to be facilitators and champions of a new curriculum which utilizes feedback, coaching, and new models of programmatic assessment.^4^^–^^6^ The transition to CBME brings with it opportunities for new positions.^2^^,^^3^ Two notable examples are the voluntary and emergent service roles of the Academic Advisor (AA) and Competence Committees member (CC). Academic Advisors, sometimes referred to as Coaches,^7^^,^^8^ synthesize the assessment data that trainees accrue and provide a holistic summary of what the trainee should focus on next. CC members adjudicate portfolios of assessment data and make determinations on which trainees are competent at the current stage of training and should be promoted to greater responsibilities at the next level.^7^ These roles are evolving in medical education and faculty leaders need to develop their own competencies in order to support trainee development in CBME.^3^^,^^9^^,^^10^ With the transition to CBME occurring worldwide, it is crucial that the physicians choosing to enter these CBME support roles have the skills and competencies to thrive in their new roles and to support trainees more effectively.

While faculty in medicine come into their physician roles having received extensive medical training, many receive little training on how to become effective coaches and teachers.^3^^,^^5^ Indeed, many physician assessors have been trained under different paradigms, where the focus was on summative and time-based assessment, rather than formative assessments and other forms of coaching.^2^^,^^4^^,^^5^ To ensure success, we believed that Queen’s faculty such as academic physicians and their community and distributed clinical preceptors entering the AA and CC members’ positions must be responsive to the specific needs of individual residents. As well, they need to be accepting of the new competencies they themselves need when taking on these new positions.

In 2014, Queen’s University set an ambitious goal to implement CBME across all 29 of its specialty training programs starting on July 1, 2017. In our view, the successful launch of CBME at Queen’s, and the continued support of its ongoing implementation, would require the development of teaching and coaching competencies that are aligned with the roles of AAs and CCs and with the larger Queen’s community. As a first step, this study’s purpose was to identify the competencies that would guide our institutions’ faculty development to ease a successful roll-out of CBME. In keeping with the community approach to transitioning the Queen’s Postgraduate Medical Education (PGME) to CBME, our goal was to create evidence-based faculty development initiatives such as workshops, small group teaching sessions, and modules) that would assist in developing these competencies. This community approach encompassed faculty, clinical preceptors and resident physicians in Royal College of Physician and Surgeons of Canada programsacross our academic and distributed medical education networks including preceptors and adjunct faculty. As a second study outcome, the two-phase survey design would be able to show if a consensus on the perceived necessary competencies could be found among the stakeholders at Queen’s. We conducted a literature review to ascertain existing competencies for AAs and CCs. PGME stakeholders completed a survey to refine and achieve relative consensus on what the competencies in the faculty development initiatives would need to be going forward. The approval of the stakeholders in the surveys would then be described and compared to show the extent to which the different stakeholders came to agree after the second iteration of the survey.

### Research questions

1. What competencies does the medical education literature posit for AA and CC members?
2. What competencies for AAs and CCs are most appropriate for our setting?

## Methods

This project received Research Ethics Board clearance from Queen’s University and was conducted in strict compliance with the consent procedures of the Queen’s University Health Sciences and Affiliated Hospitals Research Ethics Board.

**Figure 1:**
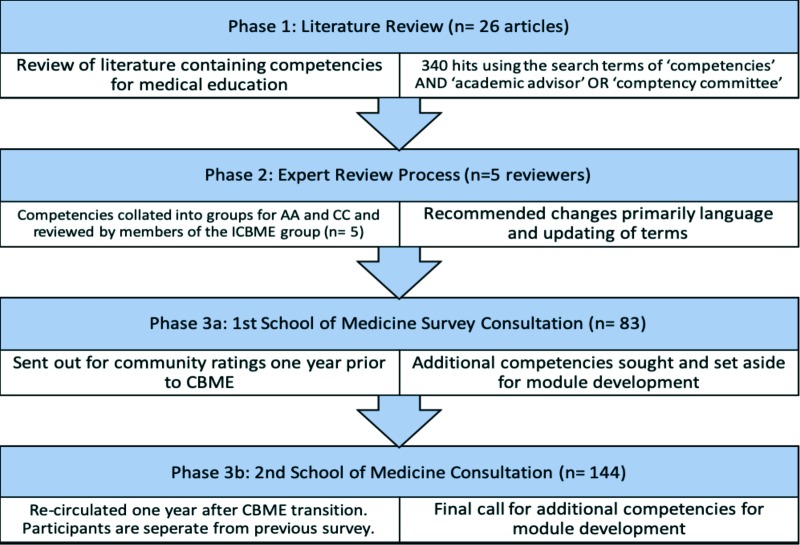
The study design and phase steps.

This project began with a literature review which informed a community-based stakeholder consultation approach comprised of two surveys delivered two years apart, which included a review of competencies by international experts involved in CBME (See Figure 1).^11^^,^^12^ Survey respondents were a varied group consisting of program directors, CBME program leads, frontline physicians, and residents involved in the transition to CBME at Queen’s University.

**Figure 2:**
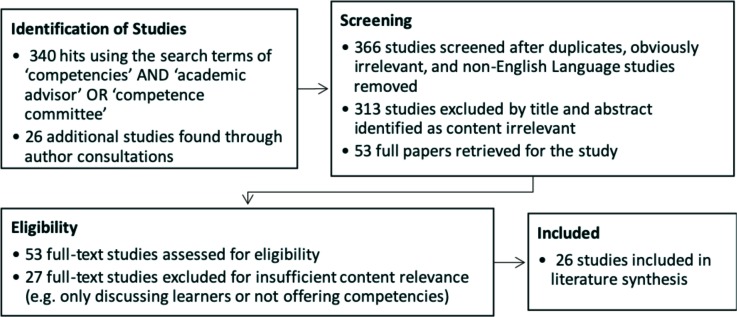
Prisma diagram for the literature review

**Phase One:** As a first step, we conducted a literature review of competencies for medical education using an all-database search of EBSCOhost in April 2016 resulting in 340 hits using the search terms ‘competencies’ AND ‘academic advisor’ OR ‘competence committee’.^13^ In addition, author consultations yielded another 26 articles for consideration (See Figure 2). As an end result, the competencies were gleaned from 26 articles that met the eligibility criteria of providing peer-reviewed, English language in-text competencies for medical educators.^3^^-^^5^^,^^9^^–^^31^

**Phase Two:** We collated competencies into itemized lists for AA and CC members and then circulated them by email to five invited members of the ICBME^37^ group (an international consortium of CBME leaders external to the author team) who proposed slight language changes and updating of terms which were reconciled by the author team before recirculating. The external reviewers stated their approval of the list as comprehensive within three iterations.

**Phase Three:** Once we finalized the list of competencies for AAs and CCs, we sought to ascertain the level of approval of the Queen’s PGME community stakeholders of the 29 Royal College Specialty programs. To this end,we administered a survey of these competencies by email listservs using Qualtrics software to stakeholder groups including residents, attending physicians, CBME leads, and program directors totalling 316 stakeholders throughout the Royal College Specialty PGME community at Queen’s one year before the transition to CBME (June 2016; n= 83; response rate = 26.2%), and one year after the transition to CBME (July 2018; n=144; response rate= 45.6%). The two survey samples were therefore gathered two years apart and were independently gathered from one another with anonymous responses.

For both survey occasions, stakeholders ranked the competencies they thought were most important using a 5-point Likert-type scale (1= strongly disagree, 5= strongly agree). Respondents were asked how important a given competency was from their perspective for the given role. This survey was designed to delineate how well received our developed competencies for AA and CC members matched with expectations of stakeholders “on the ground”, and what changes would need to be made to further align these competencies with the postgraduate community of AAs and CCs. Participants also proposed additional competencies for AAs and CCs from their own perspectives. These stakeholder-identified additional competencies which were then triangulated with those included in the survey to form the learning goals of modules aimed to support the transition for physicians entering the new positions of AA and CC members. The responses were data-cleaned and then analysed as independent samples before and after implementation of CBME in SPSS v.23 yielding descriptive and inferential statistics including analysis of variance (ANOVA). This allowed us to identify the degree of acceptance or rejection of the competencies among the school as a whole and amongst each of the various groups.

This survey was designed to delineate how well received our developed competencies for AA and CC members matched with expectations of stakeholders “on the ground”, and what changes would need to be made to further align these competencies with the postgraduate community of AAs and CCs. Participants also proposed additional competencies for AAs and CCs from their own perspectives. These stakeholder-identified additional competencies which were then triangulated with those included in the survey to form the learning goals of modules aimed to support the transition for physicians entering the new positions of AA and CC members. The responses were data-cleaned and then analysed as independent samples before and after implementation of CBME in SPSS v.23 yielding descriptive and inferential statistics including analysis of variance (ANOVA). This allowed us to identify the degree of acceptance or rejection of the competencies among the school as a whole and amongst each of the various groups.

## Results

The review and consolidation of literature findings resulted in twenty-one competencies for academic advisors and ten for competence committee members. The review of competencies by five members of the ICBME group, who are recognized as influencers within the CBME movement made primarily language revisions and gave their approval to the competencies displayed in Tables 2 and 4.

**Table 1:** Pre CBME and Post CBME demographics

	Pre-CBME	Post-CBME
Attending Physician	52	90
CBME Lead	9	9
Program Director	10	9
Resident	12	15

**Table 2:** Academic Advisor competencies ratings from one year before and one year after CBME

Proposed Academic Advisor Competencies	Pre-Mean (Variance)	Post-Mean (Variance)
Facilitates a dialogue with learner to select pertinent learning goals (e.g., program objectives) and strategies to progress	4.29(0.376)	4.77 (0.179)
Engages other supervisors in the learning plan (helps operationalize plan)	3.69(0.731)	4.77 (0.247)
Facilitates learner to take ownership of developing and updating learning plans	4.33(0.364)	4.84 (0.134)
Analyzes challenges to progression and collaborates with learner to plan specific strategies to overcome these challenges	4.29(0.432)	4.58 (0.247)
Acts as a resource for colleagues for educational problem solving in clinical training	3.5(0.741)	4.07 (0.312)
Have an in-depth understanding of the residency program’s structure and objectives of training	3.87(0.871)	4.42 (0.344)
Uses the program’s tools to help learner synthesize the different pieces of formative feedback (e.g., field notes, encounter cards, etc.)	3.79(0.907)	4.63 (0.359)
Integrates learner’s self-assessment and in-training assessments to identify appropriate learning plans	4.12(0.552)	4.41 (0.416)
Fosters and facilitates learner in taking ownership of lifelong learning	4.26(0.563)	4.44 (0.35)
Finds common ground in the case of discrepancy between learner’s self-assessments and supervisors’ in-training assessments	3.94(0.612)	4.33 (0.417)
Assists colleagues to develop lifelong learning skills in their learners	3.17(0.816)	3.55 (1.325)
Asks about, takes interest in, and explores career goals, and plans a career strategy with learner.	3.97(0.796)	4.36 (0.282)
Fosters the development of the learner’s professional identity.	3.76(0.731)	3.55 (0.19)
Demonstrate sensitivity and responsiveness to each learner as an individual, including respecting privacy, autonomy, and professional boundaries.	4.23(0.528)	4.53 (0.252)
Demonstrate sensitivity and responsiveness to learner diversity, including ability, disability, gender, age, culture, ethnicity, and sexual orientation.	4.24(0.552)	4.6 (0.291)
Invest in each learner’s growth and skill development.	4.07(0.527)	4.66 (0.196)
Are aware of competing demands on learners and learners’ personal/professional issues, which might affect their growth.	3.96(0.502)	4.61 (0.313)
Elicit each learner’s barriers to learning and work to overcome them.	4.05(0.48)	4.7 (0.262)
Recognize learners in distress and provide appropriate resources within the educational structure to assist.	4.44(0.402)	4.73 (0.197)
Seeks ongoing feedback from experienced colleagues in developing skills as an academic advisor.	3.78(0.646)	4.61 (0.289)
Participates in a community of practice or engages with others to share “best practices” in supporting learners with progression challenges.	3.65(0.79)	4.2 (0.409)

**Table 3:** Community proposed competencies for Academic Advisors

Theme of competencies	Proposedcompetencies	Example stakeholder quotes offered as competencies
Effective Communication and Mentoring	20	Communication skills to help resident develop their own self-regulation
		Excellent interpersonal skills
		Recognizes learners in difficulty
Advocate, Supportive, Approachable	14	Approachable
		Non-intimidating - Possess the qualities that would allow a resident to express their concerns or insecurities freely without fear of reprimand
		Active listener
CBME Expertise and CanMeds competencies	9	Knowledgeable - About both the program requirements and the processes of competency assessments.
		For example, many of our staff are under the impression that residency will be strictly competency (vs time-based), which is a common misperception that the Royal College has repeatedly denied.
		Knowledge of the CBME stages / EPAs and how the residents progress, in order to offer appropriate assessment of resident in their current stage.
Effective Feedback and Assessment	11	Ability to give specific feedback
		Analyzes and integrates diverse assessment data to generate comprehensive feedback
		Ability to synthesize various forms of assessment
Clinical Teaching and Learning	11	Understanding of CBME stages and evaluations
		Specialty knowledge - ie. it should be an emergency doctor for emergency resident
		Royal college certified physician in the same speciality of the trainee
Objectivity	3	Objective - Use objective, rather than subjective, measures to assess progress
		Impartial
Reliability and Organizational Skills	5	Reliable in timeliness of feedback and meeting
		Time management

**Table 4:** Competence Committees member competencies ratings

Proposed Competence Committees Competencies	Pre-Mean (Variance)	Post- Mean (Variance)
Demonstrates skill at interpreting different assessment tools	3.94 (0.601)	4.41(0.412)
Uses appropriate tools to correctly interpret the learner’s performance	4.13 (0.487)	4.49(0.365)
Collates and interprets evidence of learning and provides meaningful insight based on multiple sources, including direct observation	4.21 (0.546)	4.52(0.364)
Assists program leaders in improving assessment systems	3.77 (0.696)	4.29(0.430)
Supports implementation and enhancement of program assessment systems through feedback about program performance	3.74 (0.618)	4.47(0.308)
Understand their role, policies, and the process regarding resident assessment and progress	4.18 (0.610)	4.82(0.148)
Fosters and facilitates learner in taking ownership of lifelong learning	3.92 (0.750)	4.16(0.584)
Makes evidence-based decisions in the case of discrepancy between assessment data sources	4.13 (0.520)	3.98(0.365)
Assists colleagues to develop lifelong learning skills in their learners	3.38 (0.994)	3.43(0.827)
Distinguishes between formative and summative assessment.	3.82 (0.652)	4.49(0.370)

The newly defined competencies for AAs and CCs were well received with an overwhelmingly positive reception from all groups of stakeholders although there were varied levels of approval (See Table 1 for stakeholder demographics). Assessment, as well as mentoring competencies were the most positively rated

The highest rated competencies for AAs centred upon mentoring, such as “recognize learners in distress and provide appropriate resources within the educational structure to assist” (at 4.77 out of 5), followed by “facilitates learner to take ownership of developing and updating learning plans” (at 4.73 out of 5). While still highly rated skills, assessment competencies were rated as less important for AAs than mentoring competencies. The lowest rated competency was “assists colleagues to develop lifelong learning skills in their learners, which was rated at 3.59 out of 5 (with a variance of 1.11) indicating polarized views on the importance of this competency.

In the case of AAs (See Table 2) the level of agreement with competencies, although tentative prior to the implementation of CBME, was significantly increased at the 99% confidence level in the ANOVA (F= 26.187, p= <0.001, d= 1.22; large effect size) when surveyed after implementation. There were significant differences in approval of AA competencies between residents and groups composed of attending physicians in the pre-implementation sample (F= 4.886, p = <0.01, d= 0.83; large effect size).

Residents reported much less approval. However, at the time of the post-implementation sample, there were no significant differences and approval was consistent indicating the formation of a relatively strong consensus.

In addition to rating the competencies from the literature, raters also proposed their own competencies (See Table 3). Seventy-three additional competencies were proposed which were categorized into eight groupings Similar to the competencies identified in the literature, the most common competencies for AA involved mentoring and coaching skills.

For CC members, the highest rated competencies (See Table 4) were centred around enforcing policy and triangulating and utilizing assessment data including: “understand their role, policies, and the process regarding resident assessment and progress” (rated at 4.72 out of 5). Another very highly rated competency was “collates and interprets evidence of learning and provides meaningful insight based on multiple sources, including direct observation” (at 4.50 out of 5). Similar to the AA group, “assists colleagues to develop lifelong learning skills in their learners” was the lowest rated competency (at 3.72 out of 5). In the case of Competence Committees members (See Table 4), the level of agreement with competencies although tentative prior to CBME implementation, when surveyed after implementation levels of agreement consistently significantly increased at 99% confidence (F= 9.336, p= 0.003, d= 0.91; large effect size).

There were significant differences in approval of CC competencies between residents and groups composed of attending physicians in the pre-implementation sample (F= 3.944, p = 0.01, d= 0.60; medium effect size), however, at the time of the post-implementation sample, there were no significant differences which supports the idea that a consensus emerged among all the stakeholders

Respondents rated thecompetencies from the literature and offered their own competencies for Competence Committee members. 36 additional competencies were proposed which were categorized into six groupings (See Table 5). The most common of these additional proposed competencies were focused on developing a deep knowledge of CBME.

**Table 5:** Community proposed competencies for Competence Committees

Theme of competencies	Number of proposed competencies	Example stakeholder quotes offered as competencies
Fluency with assessment and integrating information	8	Clear understanding of competencies required at each stage
		well versed in the principles of assessment and CBME
CBME and program knowledge	9	Recognizes the roles for learning plans, remediation, and probation
		Understand CBME process for promotion to next level
		Clear understanding of competencies required at each stage
Following policy	4	Understands and follows decision making process for the CCC Advocates for resident learning
		Understand the University Appeals process
Leadership and being part of a team	7	Excellent interpersonal skills
		Collaborative with colleagues
Organized	3	Good administrative abilities
		Timely reports and recommendations
Providing direction to at-risk learners and advocacy	5	Be able to develop learning plan for residents in difficulty.
		Knowledge of the support structures in place to diagnose and assist the resident in need

## Discussion

This study identified 21 AA competencies and 10 CC competencies from the literature and refined them to the Queen’s context enabling the process of tailored faculty development that molded consensus while building faculty capacity. Although AAs are expected to help monitor trainee development to help adjudicate and deliver feedback to the residents in their care, the AA’ competencies were more highly rated when they focused on mentoring and coaching skills, rather than assessment skills. In comparison, CC members’ competencies were rated more highly when focused on assessment, integrating multiple sources of formative and summative assessments, and abiding by the new policies governing CBME such as when to promote a resident (in addition to the actions of past resident promotion committees which operated with less frequent assessments typically of a summative nature). As a result of the competencies most highly rated by the Queen’s raters, modules were developed by CBME content experts with the competencies as learning goals. The modules are a central part of the induction process for faculty new to CBME, constituting a key part of the portfolio of new faculty resources at our institution.

The community-based approach of refining, proposing, and rating competencies was important as it promoted engagement from all stakeholders including faculty, residents, and CBME specialists across PGME. It also provided a comprehensive view on what the faculty in these AA & CC positions needed to be able to do, which was informed by the literature, molded by expert consensus, and uniquely aligned to current practices of medical education delivery at Queen’s University. Following analysis of survey results, the final modules were assigned to experts who began development on online modules available to all PGME faculty. These modules will take the form of slideshows that will be narrated and presented using an online learning management software to be accessible publicly as a part of the portfolio of efforts to facilitate the development of skilled AAs, CC members, and preceptors in general.

Raters had varying expectations for what successful AA and CC members should be able to do before implementation. After implementation, the gap had closed for both the AA and CC competencies. The events of implementation, chiefly the mandated faculty development for program leaders and consistent outreach efforts with our larger medicine community convincingly paid dividends in terms of increased approval of the competencies and molding consensus on the expectations for AAs and CCs at our institution. The lack of differences after implementation points towards a greater degree of shared understanding among stakeholders and lends support to the notion that taking an accelerated path together as an institution, as was done at Queen’s, can result in a culture shift towards shared priorities.^10^

## Limitations

This study has limitations in generalizability and methodology. This was a single centre study, which means that it is as much a reflection as a product of Queen’s University. Although, other institutions would likely face similar challenges in infrastructure, capacity, stakeholder wariness, and structural change when faced with the same paradigm shift, other contextual factors are necessary to consider when generalizing these findings to other contexts. Methodologically, due to the ethical concerns of potentially identifying stakeholders, the survey asked respondents to answer limited demographic questions, which prevented comparisons by some demographic factors. The anonymous nature of responses also made it impossible to note which respondents answered both surveys. This study focused on institutional perspectives rather than intergroup comparisons, making the gathered demographic information sufficient.

### Conclusion

We found value in taking an active community-based approach to identifying the competencies that would guide our institutions faculty development. The creation of CC members and AA roles requires a sustained investment of specialized professional development. To this end, our evidence-informed approach was an effective way to develop shared competencies for teaching faculty that enriched our community of practice and developed a better understanding of each program’s needs.

### Practice points

- AA’s competencies were most aligned with mentoring and teaching rather than assessing
- The lack of consensus very much present one year before implementation in terms of priorities has largely subsided one year after implementation as groups work together in the new reality of CBME as opposed to being driven by their initial conceptions
- CC members’ competencies were most aligned with assessment knowledge and less with a teaching role
- Communities transitioning to CBME can greatly benefit from engaging their stakeholders in the design of professional development to best deliver content that suits the community’s needs.
